# Direct Observation of Single Amyloid-β(1-40) Oligomers on Live Cells: Binding and Growth at Physiological Concentrations

**DOI:** 10.1371/journal.pone.0023970

**Published:** 2011-08-25

**Authors:** Robin D. Johnson, Joseph A. Schauerte, Kathleen C. Wisser, Ari Gafni, Duncan G. Steel

**Affiliations:** 1 Department of Biophysics, The University of Michigan, Ann Arbor, Michigan, United States of America; 2 Department of Biological Chemistry, The University of Michigan, Ann Arbor, Michigan, United States of America; 3 Department of Physics, The University of Michigan, Ann Arbor, Michigan, United States of America; 4 Department of Electrical Engineering and Computer Science, The University of Michigan, Ann Arbor, Michigan, United States of America; National Institute on Aging Intramural Research Program, United States of America

## Abstract

Understanding how amyloid-β peptide interacts with living cells on a molecular level is critical to development of targeted treatments for Alzheimer's disease. Evidence that oligomeric Aβ interacts with neuronal cell membranes has been provided, but the mechanism by which membrane binding occurs and the exact stoichiometry of the neurotoxic aggregates remain elusive. Physiologically relevant experimentation is hindered by the high Aβ concentrations required for most biochemical analyses, the metastable nature of Aβ aggregates, and the complex variety of Aβ species present under physiological conditions. Here we use single molecule microscopy to overcome these challenges, presenting direct optical evidence that small Aβ(1-40) oligomers bind to living neuroblastoma cells at physiological Aβ concentrations. Single particle fluorescence intensity measurements indicate that cell-bound Aβ species range in size from monomers to hexamers and greater, with the majority of bound oligomers falling in the dimer-to-tetramer range. Furthermore, while low-molecular weight oligomeric species do form in solution, the membrane-bound oligomer size distribution is shifted towards larger aggregates, indicating either that bound Aβ oligomers can rapidly increase in size or that these oligomers cluster at specific sites on the membrane. Calcium indicator studies demonstrate that small oligomer binding at physiological concentrations induces only mild, sporadic calcium leakage. These findings support the hypothesis that small oligomers are the primary Aβ species that interact with neurons at physiological concentrations.

## Introduction

Soluble oligomeric forms of the 39-to-42 residue amyloid-β peptide, the primary component of Alzheimer's disease plaques, may be a key player in synaptic dysfunction and cell death [1]–[3]. However, studying this system at physiological peptide concentrations presents challenges. Soluble Aβ is only detected at nanomolar to picomolar levels in the human brain [4], [5] which renders study at physiological concentrations by traditional biochemical methods problematic. Efforts to pinpoint the neurotoxic aggregates have also been complicated by the finding that at physiological concentrations, Aβ exists as a mixture of metastable species [6]–[8]. Additionally, Aβ-membrane interactions are complex and variable; externalized Aβ may bind to specific cellular receptors or protein complexes (e.g. NMDA receptors [9], [10] and α7 nicotinic acetylcholine receptors [11]), associate with phosphatidylserine in the membrane [12], or bind to and insert directly into the lipid bilayer [13]–[15].

Due to the complexities of this system, the primary mechanism by which soluble Aβ induces toxicity remains unclear. Increased oxidative stress, interference with synaptic transmission, and impaired axonal transport may contribute to the death of affected neurons [1], [16]. According to one strongly supported hypothesis, Aβ initiates toxic effects by disrupting membrane integrity, either by direct formation of pores in neuronal cell membranes [14], [17], [18] or by carpeting the membrane, resulting in thinning of the lipid bilayer and loss of ion homeostasis [19], [20]. The involvement of oligomeric Aβ in toxicity is more strongly established. Levels of various soluble, oligomeric forms of Aβ have repeatedly been shown to be a stronger indicator of disease state in humans than plaque load [4], [5], [21], [22]. Stabilized versions of dimers reduce long-term potentiation in cultured neurons [23]. Experiments with cross-linked oligomers have shown that neurotoxicity increases nonlinearly with oligomer size, comparing dimers, trimers, and tetramers [24]. Finally, in one widely cited study, chemically purified oligomers were shown to induce instantaneous calcium leakage in cultured neuroblastoma cells [19].

If toxicity is indeed mediated primarily by extracellular Aβ, as many of these studies suggest, binding to some component of the cell membrane is a necessary step in the pathway to functional abnormalities. Understanding the mechanism of binding for living cells exposed to physiological Aβ concentrations requires novel approaches. Chemically stabilizing various Aβ aggregates in solution may drastically alter normal aggregation rates [25] and membrane-binding behavior, obscuring mechanistic details. Furthermore, the harsh solvents used in certain of these procedures (e.g. hexafluoroisopropanol) may in themselves damage cell membranes, further complicating the problem [26]. A number of groups have begun to study Aβ-membrane binding on live cells using fluorescence-based techniques. Using confocal microscopy, Bateman, et al. monitored the formation of fluorescently-labeled amyloid-β(1-42) aggregates on living PC12 cells [27]. At low micromolar concentrations, they observed formation of two distinct types of large membrane-bound aggregates. More recently, Nag et al. have studied FITC-labeled Aβ40 bound to cell membranes after several minutes' exposure to near-physiological Aβ concentrations [28]. Fluorescence correlation spectroscopy and fluorescence lifetime measurements were used to characterize the mobility and membrane insertion of the peptide, but no specific cell-bound oligomers were identified.

Previously, our group has utilized single molecule TIRF (Total Internal Reflection Fluorescence) microscopy to study the size distribution of amyloid-β(1-40) (hereafter referred to as Aβ40) oligomers formed in solution at physiological concentrations [8]. We also recently used single molecule intensity measurements to examine which Aβ40 oligomers correlate with conductance changes on a model membrane [29]. Here we present single molecule data showing that small Aβ40 oligomers formed in solution bind directly to living SH-SY5Y neuroblastoma cells at physiological concentrations. These oligomers, ranging in size from dimers to hexamers and larger, are relatively immobile and widely distributed on the cell body. Using slide-localized oligomers as a cross section of the species present in solution, we compare the sizes of cell-bound aggregates to the distribution observed for Aβ40 in solution. We find that cell-bound oligomers include a small proportion of distinctly larger (hexameric and greater) oligomers, demonstrating that oligomers may grow or colocalize following binding to the cell membrane.

## Results

### Comparison of freshly dissolved HL647cAβ40 and unlabeled Aβ40 by gel filtration HPLC

In order to examine the cell membrane binding behavior of Aβ40, nanomolar concentrations of Aβ40, fluorescently labeled at the N-terminus, were applied to SH-SY5Y neuroblastoma cells. The N-terminus of Aβ is solvent-exposed and therefore unlikely to be involved in the β-sheet formation which drives fibrillization [30], [31]. Also, work done in our laboratory and others indicates that various forms of N-terminally labeled Aβ40 behave similarly to unlabeled Aβ40 in terms of fibrillization [32], ability to permeabilize synthetic membranes [8], [29] as well as rat basophilic leukemia cell-derived membrane blebs (Figure S1), and toxicity to cultured cells [33]. The current studies were performed using Aβ40 labeled with HiLyte Fluor 647 by a C2-maleimide linkage at the N-terminus (termed HL647cAβ40). To confirm that this peptide behaves similarly to the unlabeled peptide on solubilization, we ran freshly prepared HL647cAβ40 and unlabeled Aβ40 on HPLC. Both versions of the peptide elute primarily as a single peak with dimer-to-trimer molecular weight (Figure S2).

### HL647cAβ40 at physiological concentrations rapidly binds to SH-SY5Y neuroblastoma cells

To examine the questions of how Aβ40 binds to cell membranes and what species are present following binding, we applied HL647cAβ40 to live neuroblastoma cells at near physiological peptide concentrations and measured the integrated intensity of cell-bound particles. Cells were imaged following a 10 minute exposure to 50 nM HL647cAβ40 in media; in order to obtain cross-sections through the cell membranes, the objective was focused at a z-plane that fell halfway between the apical and basal portions of the cell membrane.

**Figure 1:**
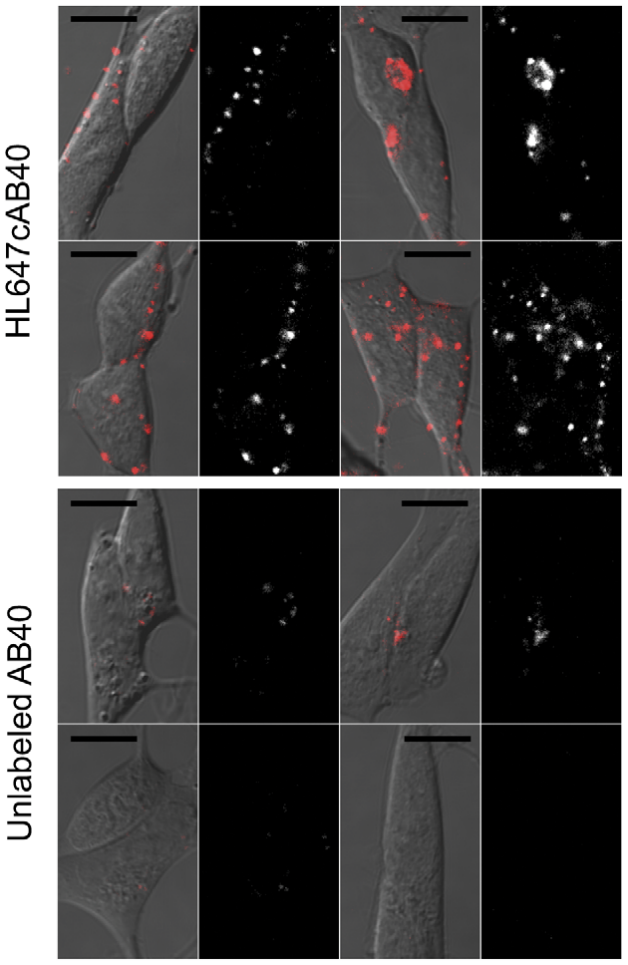
Single HL647cAβ40 oligomers bind to live neuroblastoma cells within minutes at 50 nM. Single-molecule sensitivity confocal images of cells treated with 50 nM HL647cAβ40 or unlabeled Aβ40 for 10 minutes. Differential Interference Contrast images with HL647 channel fluorescence overlaid in red are shown to the left of the corresponding HL647 channel only images. Scale bars, 10 µm.

Representative images of cells exposed to HL647cAβ40 and unlabeled Aβ40 (Figure 1) reveal both internal and surface-localized fluorescent particles in the HL647cAβ40 samples. Some form of membrane binding is believed to be a key step in toxicity directly mediated by Aβ40, and while several groups have observed internalization of fluorescently labeled Aβ40 and Aβ42 by neuroblastoma cell lines [33], [34], only one has reported binding to live cells at physiological concentrations [28]. During analysis, we focused on measuring the size of those particles that were edge-localized (as this is where membrane-bound oligomers could be most clearly identified).

### Freshly prepared HL647cAβ40 solutions contain primarily monomers and dimers

**Figure 2:**
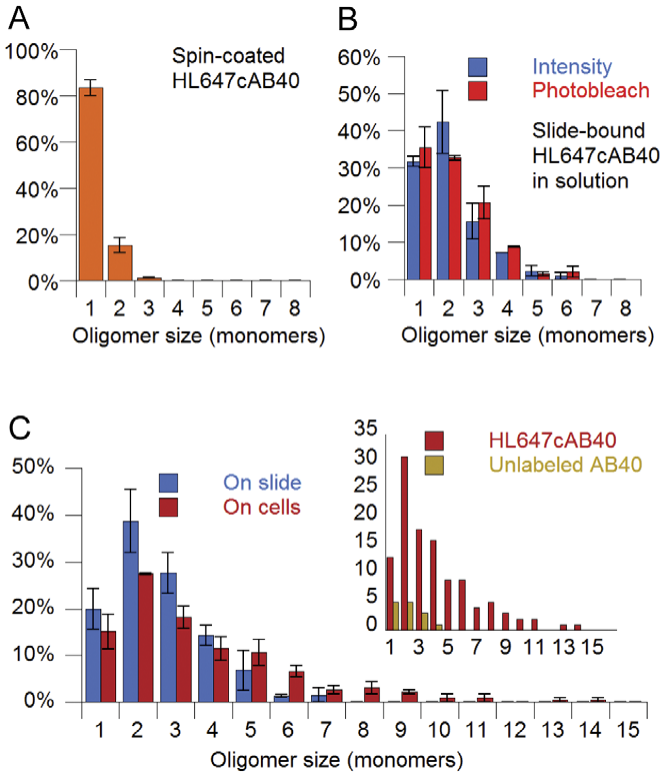
Small HL647cAβ40 oligomers form in solution and grow or cluster following binding to cells. (A) Normalized oligomer size distributions for freshly solubilized HL647cAβ40 diluted to 0.5 nM in 10 mM phosphate buffer and spin-coated onto a kilned glass slide (*orange, N = 70 and 91 particles*). Size was measured by counting photobleach steps in sequences of confocal scans. Error bars represent the standard deviation for 2 experiments. (B) HL647cAβ40 was diluted to 50 nM in media, bound a kilned glass slide, and imaged on the slide in solution, without cells present Size was measured by confocal scan integrated intensity (*blue, N = 112 and 112 particles)* or by TIRF-mode single molecule photobleach step count (*red, N = 100 and 117 particles*). (C) Normalized oligomer size distributions observed for HL647cAβ40 diluted to 50 nM in media and bound to the slide in samples containing cells (*single-hatched, N = 119 and 122 particles*) and bound to cells (*filled-in, N = 114 and 112 particles*). Inset: Raw oligomer size distributions obtained when the analysis protocol is performed on similar numbers of cells treated with labeled (*red, N = 60 frames, 117 particles detected*) and unlabeled Aβ40 (*yellow, N = 63 frames, 14 particles detected*). Both distributions were obtained using confocal mode integrated intensity.

To study how Aβ40 oligomerization and binding occur at physiological concentrations, we wished to begin with monomeric Aβ40. We therefore examined the size distribution of oligomers present in freshly dissolved HL647cAβ40 samples by recording photobleach trajectories on dry, spin-coated peptide (Figure S3; Figure S4). Of spots with clean photobleach traces, 83% ±3% bleach as monomers and 15% ±3% bleach as dimers (Figure 2A). These results led us to conclude that the majority of freshly dissolved HL647cAβ40 is monomeric or dimeric.

### Fluorescence intensity calibration enables determination of oligomer stoichiometry in the confocal mode

To measure oligomer size on living cells, we developed a protocol to correlate particles' fluorescence intensity values with the number of Aβ40 monomers they contain. When laser power is below saturation, the fluorophore emission varies linearly with excitation power. Under these conditions, the slope of total intensity from a given volume versus the number of molecules present in the volume yields intensity-per-molecule. The fluorescence intensity of an oligomer can be divided by this value to yield the number of Aβ40 monomers present in the oligomer (assuming the quantum yields of the free dye and peptide-bound dye are the same, as discussed below).

To determine the intensity of a single HL647 dye molecule in solution, a series of confocal scans were performed on various dilutions of HL647 hydrazide. The average intensity of a 1.6 µm by 1.6 µm by 1 µm volume element was measured at 10 nM HL647 hydrazide concentration increments from 0 nM to 60 nM (0 to 102 dye molecules per volume element). In this regime, intensity per number of molecules in a volume element is linear, with a best-fit correlation coefficient of 0.996558. The slope of this line represents an intensity-per-molecule value that can be used to extract HL647cAβ40 oligomer size (Figure S5).

The validity of this calibration method requires the assumption that the fluorescence yields of HL647cAβ40 and free HL647 hydrazide in solution are identical. Collisional quenching from the attached peptide is one mechanism that might reduce the efficiency of photon emission of the fluorophore in HL647cAβ40. Fluorescence lifetime measurement was used to eliminate this possibility. For dynamic quenching, the ratio of intensity of the quenched fluorophore (*I_q_*) to that of the unquenched fluorophore (*I_0_*) is equal to the ratio of the fluorophore lifetimes (*τ_q_* and *τ_0_*, respectively) under each condition [35]:
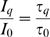
(1)


Accordingly, fluorescence lifetimes of HL647 hydrazide and HL647cAβ40 were measured in imaging buffer. Fitting the fluorescence decay curves using the Exponential Series Method yielded lifetimes of 1.55±0.24 ns for the free dye and 1.64±0.09 ns for HL647cAβ40. Hence, the two lifetimes are equivalent within experimental error. Collisional quenching from Aβ40 does not reduce the quantum yield of the dye in HL647cAβ40.

Other factors may also affect the relative fluorescence efficiencies of the free dye and the peptide-bound dye, when HL647cAβ40 aggregates or associates with surfaces. One possibility is that oligomerization of the HL647cAβ40 peptide, its insertion into a biological membrane, or its interaction with a surface results in significant quenching. However, we have previously addressed these questions using Aβ40 N-terminally labeled with HiLyte Fluor 488 and established that oligomers up to 20 monomers in size have fluorescence decays that fit well to a single exponential, and fluorescence lifetime is not altered for aggregates of this size even after binding to (and insertion in) lipid bilayers [29]. As HL647cAβ40, like HL488Aβ40, has an N-terminally located fluorophore, it is reasonable that low-order HL647cAβ40 oligomers also retain the lifetime of the dye under these conditions.

To further verify the accuracy of this method, a distribution generated with our calibration was compared to one compiled from photobleach trajectories taken on a total internal reflection fluorescence (TIRF) microscope. Samples for both measurements were prepared by dilution of HL647cAβ40 to 50 nM in media over a kilned, autoclaved glass slide, which results in oligomers binding to the slide (Figure S3; Figure S6). The oligomer size distributions obtained using the two methods were highly similar (Figure 2B). In both samples, approximately 70% of oligomers fall in the monomer-to-dimer range, with roughly 30% trimers to hexamers. These distributions differ substantially from that described above for freshly prepared peptide (98% monomer/dimer); however, they do resemble those presented in the literature upon for Aβ40 in media or physiological buffers at nanomolar concentrations [6]–[8], [36]. We note that unlike our earlier studies on dried samples [8], where we demonstrated significant variations in dipole orientation between monomers in a given oligomer, these measurements were made in solution. The data shows that this change allows orientational motion of the fluorophore emission dipoles, leading to the same time-averaged intensity for each monomer in an oligomer. The equal magnitudes of the photobleach steps for oligomers in solution observed here (Figure S6) support this assumption.

### Small oligomers form in solution and bind to the membranes of living SH-SY5Y cells at physiological concentrations

Even a short incubation in media at 37°C results in some formation of oligomers in the trimer-to-hexamer size range (Figure 2B). Comparing the size distribution obtained for on-cell oligomers with the on-slide distribution (Figure 2C) reveals a small decrease in the numbers of dimers and trimers observed on cells. Conversely, particles with intensities corresponding to oligomer sizes from heptamers to 14-mers comprise a significant component of the on-cell distribution (10.6%), while the largest measurable oligomers observed on slides are heptamers (1.5% of the total on-slide distribution). The oligomer size distribution on the cell surface is thus shifted towards larger aggregates. (Note: These histograms exclude rarely detected spots with full width at half-maximum exceeding 250 nm and particles saturating the detector at more than three pixels—20-mers and larger by integrated intensity).

A prominent concern in live-cell single molecule microscopy involves signal contamination by autofluorescence. As an autofluorescence control, we performed our analysis on cells treated with unlabeled Aβ40. Figure 2C (inset) shows that the vast majority of detected spots in the HL647cAβ40 sample indeed represent fluorescently labeled peptide. Additionally, all of the autofluorescent spots detected by our analysis in the unlabeled Aβ40 sample have integrated fluorescence intensities that place them in the monomer-to-tetramer range.

### Cell perimeter-localized HL647cAβ40 oligomers are membrane-bound and susceptible to potassium iodide quenching

**Figure 3:**
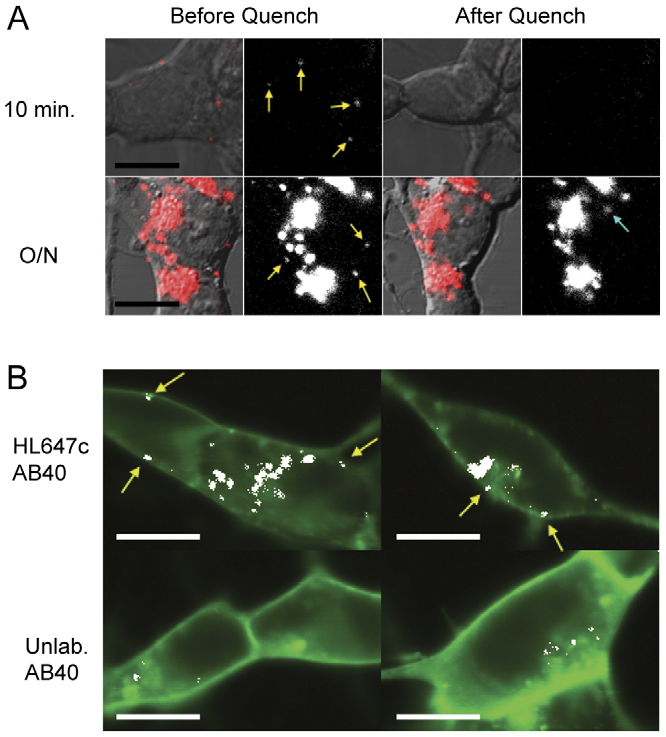
Cell-bound Aβ is quencher-accessible and colocalizes with a membrane marker. (A) Colocalization images of cells treated with 50 nM labeled or unlabeled Aβ40 for 10 minutes prior to labeling with the lipophilic membrane marker, DiO. HL647 fluorescence is shown in red, DiO fluorescence in green, and pixels where the two are colocalized in white. Edge-localized fluorescent particles colocalizing with DiO are marked with yellow arrows. Scale bars, 10 µm. (B) Cells treated for 10 minutes or overnight with 50 nM HL647cAβ40 were imaged before and 2 minutes after addition of 300 mM potassium iodide. HL647cAβ40 oligomers that quench are marked with yellow arrows. New fluorescence spot (marked with teal arrow) presumably represents an endosome containing HL647cAβ40 which has migrated into the image plane during the incubation period. Scale bars, 10 µm.

Potassium iodide quenching studies were performed to determine whether edge-localized HL647cAβ40 oligomers were solvent-accessible. Iodide, a well-known collisional quencher, has formerly been used to characterize live cell-associated Aβ40 aggregates [27]. We imaged cells treated for 10 minutes with 50 nM HL647cAβ40 before and after the addition of 300 mM potassium iodide (Figure 3A). Under these conditions, cell-perimeter localized peptide was highly susceptible to quenching. Interestingly, the majority of large internalized aggregates in cells exposed to 50 nM peptide overnight did not quench. Subsequent experiments verified that potassium iodide is able to permeate cells and quench fluorophores localized to the cytoplasm (see Figure S7). Late stage aggregate quenching resistance may represent a fundamental change in aggregate structure [27] or an inability of iodide ions to permeate the endosome-lysosome system.

We also stained cell membranes with a lipophilic membrane dye, DiO, and performed colocalization studies. The vast majority of the HL647cAβ40 signal is colocalized with DiO (Figure 3B), suggesting that HL647cAβ40 in and on the cell is nearly all membrane-associated, bound to membrane-associated proteins, or enclosed in membrane-bound vesicles.

### HL647cAβ40 and unlabeled Aβ40 induce minimal calcium leakage

**Figure 4:**
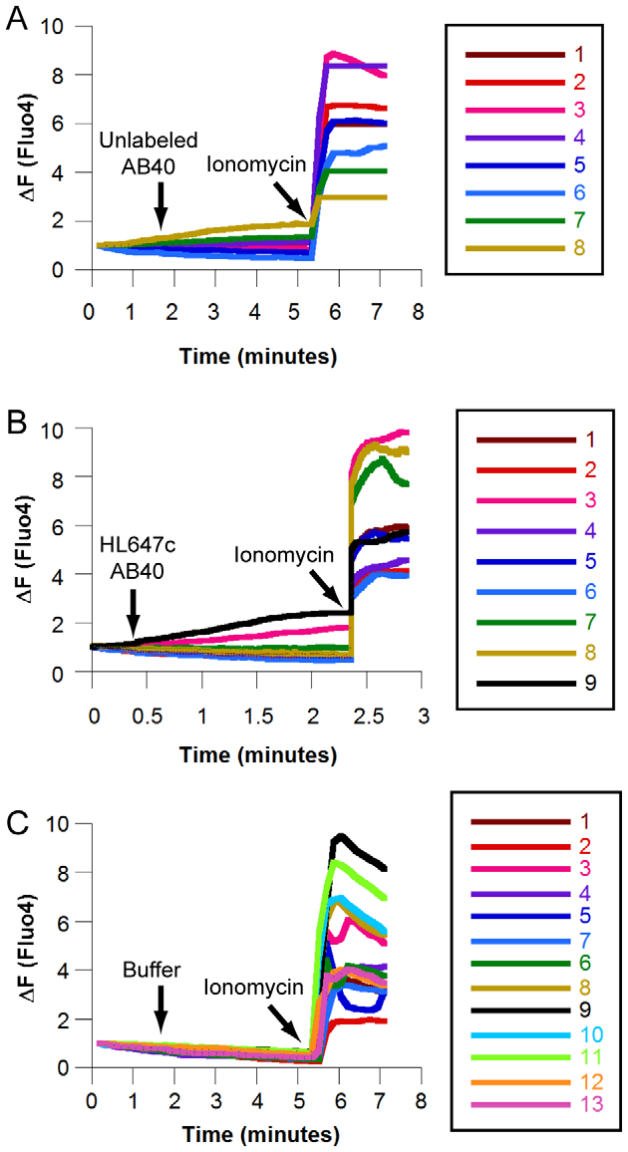
Physiological Aβ40 levels induce gradual, low level calcium leakage in a small number of cells. (A) Fluo4 fluorescence intensity plot for 8 cells treated with unlabeled Aβ40 and imaged by sequential confocal scans. Unlabeled Aβ40 was added to 50 nM after 1 minute and 40 seconds; ionomycin was added to 6 µM after 5 minutes and 20 seconds. (B) Plot for 9 cells treated with HL647cAβ40 and imaged in epifluorescence. HL647cAβ40 was added to 50 nM after 25 seconds; ionomycin was added to 6 µM after 2 minutes and 20 seconds. (C) Plot for 13 cells treated with vehicle and imaged by sequential confocal scans. 10 mM sodium phosphate was added to 50 nM after 1 minute and 40 seconds; ionomycin was added to 6 µM after 5 minutes and 20 seconds.

Application of either extracellular Aβ at supraphysiological concentrations or of chemically prepared oligomers [17], [19] has previously been shown to result in dramatic calcium influx (3-fold or greater increases in calcium indicator fluorescence), and this effect was observed within seconds in a majority of cells. To test whether freshly prepared Aβ permeabilized cell membranes to calcium at physiological Aβ levels, SH-SY5Y cells were loaded with the fluorescent calcium indicator Fluo4-AM and exposed to 50 nM Aβ. The calcium ionophore ionomycin [19] was used as a permeabilization control. Only a small proportion (roughly 10%) of cells treated with unlabeled Aβ40 (Figure 4A) or HL647cAβ40 (Figure 4B) exhibited any increase in fluorescence as compared to controls (Figure 4C). Observed fluorescence increases were less than 2-fold and occurred gradually over the course of 3 to 5 minutes.

## Discussion

Physiologically meaningful experiments on Aβ binding to live cells and the resulting toxicity have historically been difficult for reasons previously discussed. Emerging single molecule techniques represent one avenue for overcoming these barriers. Here, we adapt a conventional confocal laser scanning microscope to perform single molecule measurements on slide-localized and cell-bound single Aβ40 oligomers. By optimizing our imaging parameters, we identified a linear regime in which integrated intensity level for a given volume element was directly proportional to number of molecules present. We confirmed the accuracy of this method by another technique commonly used for measuring oligomer size, total internal reflection fluorescence (TIRF) single molecule photobleaching [37], [38].

Using this calibration, we find that HL647cAβ40 rapidly forms oligomers in the trimer to hexamer range upon dilution to 50 nM in physiological buffers and exposure to surfaces. Importantly, our treatment did not utilize harsh solvents, unnaturally high peptide concentrations, or chemical modification of oligomer structure, and our initial freshly prepared Aβ40 samples contain over 80% monomers (Figure 2A). SH-SY5Y neuroblastoma cells exposed to 50 nM HL647cAβ40 both bind the peptide and internalize it, as previously reported [33], [34]. Binding to the membrane or membrane-associated structures is a prerequisite for internalization, and if membrane-binding of a specific oligomer population were found to correlate with toxicity, such a species (or its binding site on the membrane) could be specifically targeted. Accordingly, we limited our quantitative study of cell-associated HL647cAβ40 to cell perimeter-localized particles.

Significantly, cell-bound HL647cAβ40 oligomers include a small number of aggregates (roughly 10%) that are greater in size than the largest particles present on slides (Figure 2C). Decreases of approximately 20% in both monomers/dimers and trimers/tetramers are observed on cells as compared to the on-slide data. These decreases reflect a 140% increase in pentamers/hexamers and an 8-fold increase in heptamers to 14-mers. The largest “oligomers” may simply be smaller aggregates clustered in specific, high-density peptide binding domains. Alternatively, these structures may represent large oligomers formed from the on-membrane association of several slow-moving, smaller oligomers or from addition of single monomers to bound oligomers. Our recent data indicates that at low (2 nM) Aβ40 concentrations, oligomers form slowly on supported anionic lipid bilayers from self-association of diffusing monomers. However, at 100 nM, large oligomers appear much more quickly (within 2 hours), which may indicate that small oligomers binding directly from solution can recruit other species both from solution and from on the membrane (Hao Ding, unpublished observations).

We found no evidence for a diffusing, uniformly distributed population of monomers and dimers. However, if the binding of mobile labeled monomers to the membrane surface is nonuniform, or densities are lower than approximately 1 monomer per 3 µm^2^, our analysis will not detect such monomers above cellular autofluorescence. Nag et al. recently reported the presence of diffusible, membrane-bound fluorescein-labeled Aβ40 on PC12 cells exposed to near-physiological peptide concentrations [28]. Our laboratory has also observed uniformly bound pools of diffusible Aβ40 at densities up to 5 to 8 monomers per µm^2^ on synthetic lipid bilayers [29]. This disparity may result from our use of a different cell line and a lower concentration of Aβ40 than Nag et al. (50 nM vs. 150 to 350 nM) and from differences in membrane composition between living cells and model membranes.

Occasional very high-intensity fluorescent structures are visible in our images (see Figure 1). These probably represent peptide aggregates containing many more than 20 monomers. Such large species have not been rejected as possible mediators of Aβ toxicity. However, given the relative rarity of these species and the lack of obvious morphologic abnormalities in the cells to which they are bound, we have focused our analysis on oligomers of measurable size.

The majority of cell-bound oligomers are immobile on a time scale of several seconds. This immobility is likely related to Aβ40 binding or insertion sites on the membrane, a number of which have been suggested in the literature [9], [11], [12], [39], [40]. Aβ oligomers may bind to specific lipid microdomains, such as regions enriched in externalized phosphatidylserines [12], cholesterol-rich regions [39], or lipid rafts [40]. Whether such mechanisms would preclude detectable diffusion of cell-bound Aβ is unclear. Lipid microdomains are dynamic nanoscale structures [41], with lipid confinement times in the tens to hundreds of milliseconds. High-affinity Aβ40 binding to such sites may stabilize microdomains, possibly by interacting with intracellular anchor proteins. Another likely explanation is that oligomers bind to or associate with specific membrane-integral proteins or receptors [9], [11], [23], [42] that are temporarily restricted to specific locations within the cell membrane [43], [44], immobilizing the Aβ within an area small enough that positional fluctuations are undetectable. Several of these receptors are expressed in SH-SY5Y cells and could be the binding sites of the small oligomers we observe on cells (e.g. NMDA NR1 [45], α7nAChR [46], and EphB2 [47]).

Substantial evidence supports the hypothesis that Aβ-induced toxicity stems from the formation of calcium-permeable Aβ pores in cell membranes. Interestingly, treatment of SH-SY5Y cells with 50 nM fresh unlabeled Aβ40 or HL647Aβ40 results in minimal, sporadic calcium leakage (Figure 4). Superficially, these findings may seem contrary to the massive, immediate calcium influxes observed by other groups [17], [19], [48]. However, such studies have in general been performed at micromolar Aβ42 concentrations [12], [17] or using Aβ42 prepared with solvents known to destabilize membranes [19], [26]. Our own studies of Aβ40-liposome interactions indicate that membrane binding and permeabilization occur in separate stages and depend on distinct membrane characteristics [15]. The low-level calcium leakage seen here may simply reflect how extracellular Aβ40 interacts with healthy cell membranes at physiological levels. In an effort to address these complexities, this work represents a detailed analysis of the oligomeric size of Aβ species capable of binding to living cells at physiological concentrations.

## Materials and Methods

### Amyloid-β(1-40) Preparation

Synthetic unlabeled amyloid-β(1-40), HiLyte Fluor 647-labeled amyloid-β(1-40) (HL647cAβ40) and HiLyte Fluor 647 hydrazide were from Anaspec. HL647cAβ40 was synthesized with an additional N-terminal cysteine residue, to which HiLyte Fluor 647 was attached by a C2 maleimide linkage. Amyloid-β (Aβ) peptides were dissolved in 1% NH_4_OH at 0.1 mg/mL and vortexed for 30 seconds. Peptides were then lyophilized and stored at −20°C. To prepare fresh Aβ, single aliquots were dissolved in 10 mM sodium phosphate buffer, pH 7.4, at a concentration of 1 to 2 µM and pipetted 5 to 8 times to mix. Freshly prepared Aβ was used within 30 minutes to 1 hour.

### Cell Culture

SH-SY5Y neuroblastoma cells (ATCC) were maintained in phenol-red free 1∶1 DMEM/Ham's F12 (Invitrogen) supplemented with 10% fetal bovine serum (Invitrogen) and 200 units/mL penicillin/streptomycin (Invitrogen). Prior to plating, 25 mm circular No. 1 cover glasses (Fisher Scientific) were kiln-baked at 500°C for 2 hours and then autoclaved. For live-cell imaging, cells were plated onto coverslips at a density of 28,000 cells per cm^2^ and imaged 2 to 4 days following plating.

### Overcoming Autofluorescence

Initial experiments using amyloid-β(1-40) N-terminally labeled with HiLyte Fluor 488 revealed that autofluorescence of this cell line in the FITC channel was high enough to prevent definitive detection of single molecules on the cell membrane. Comparison of cellular autofluorescence levels at multiple wavelengths and of the fluorescence characteristics of available probes led us to identify the near infrared region of the spectrum as optimum for single molecule experiments. Kiln-baking slides prior to plating, imaging on days 2 to 4 following plating, and use of phenol-red free media were also key to reducing autofluorescent background.

### HPLC

Gel filtration chromatography was performed at 23°C on a Shodex PROTEIN KW-802.5 size exclusion column. Injections of 20 µL of 8 µM labeled and unlabeled Aβ40 were run on the column in 10 mM sodium phosphate, 100 mM sodium chloride, pH 7.4, at a flow rate of 1 mL/minute. The column was calibrated for molecular weight under these conditions with the following protein standards: thyroglobulin (660 kDa), aldolase (158 kDa), bovine serum albumin (66 kDa), ovalbumin (43 kDa), peroxidase (40.2 kDa), adenylate kinase (32 kDa), myoglobin (17 kDa), RNAse A (13.7 kDa), and cyanocobalamin (1.35 kDa).

### Measurement of Fluorescence Lifetimes

Fluorescence lifetime measurements were performed on HiLyte Fluor C2 maleimide-labeled Aβ40 (HL647cAβ40) and HiLyte Fluor 647 hydrazide, as described [29]. Briefly, peptide and dye were dissolved to approximately 800 nM (as measured by absorption) in 10 mM sodium phosphate buffer, pH 7.4, and diluted to 200 nM in Hanks' Balanced Salt Solution (HBSS) (Invitrogen). Reverse-mode time-correlated single photon counting measurements were then performed. Data were analyzed by the Exponential Series Method in TimeMaster software from Photon Technology International.

### Spin-Coated Sample Preparation

Freshly solubilized HL647cAβ40 was diluted to 0.5 nM in 10 mM sodium phosphate, pH 7.4, and a droplet of this was spin-coated onto a kilned glass slide. A series of 120 confocal microscopy scans were then taken with the objective lens focused at the surface of the glass slide, and the intensity of each surface-bound fluorescent particle was plotted versus scan number.

### On-slide, in Solution, and On-cell Sample Preparation

Prior to imaging, cells on coverslips were exposed to 50 nM HL647cAβ40 or unlabeled Aβ40 for 10 minutes at 37°C. Freshly dissolved peptide was added directly into media and pipetted over coverslips twice to mix. Following peptide exposure, coverslips were washed three times in Hanks' Balanced Salt Solution (HBSS), placed into a sampleholder and covered in 1 mL of HBSS for imaging.

### Total Internal Reflection Fluorescence (TIRF) Microscopy

Single molecule TIRF microscopy was performed on an Olympus IX-71 inverted microscope. A HeNe red laser (Uniphase) was focused onto the back focal plane of a 60x, 1.45 NA Olympus PlanAPO TIRFM objective; laser power at the plane of the coverslip was measured at 300 µW. Through-the-objective TIRF was performed by translation of a mirror just upstream of the objective lens. A multi-band pass SEMRock dichroic mirror was used to separate excitation from emission signal; a 620/60 excitation band pass filter (Chroma Technology Corporation) and 700/75 emission filter (Chroma Technology Corporation) were included in the setup. Images were acquired on a back-illuminated Ixon EMCCD camera, model DV887ACS-BV (Andor) and analyzed in ImageJ.

### Epifluorescence Microscopy

Epifluorescence images for selected calcium indicator studies were acquired on the same microscope as was utilized for TIRF studies, using the 488 nm line of an Argon laser (Coherent) and a FITC filter cube.

### Fluorescence Intensity Calibration

Fluorescence intensity per dye molecule was obtained by imaging free HL647 hydrazide in HBSS at concentrations ranging from 0 nM to 60 nM. These scans were acquired with the same settings as were utilized for live-cell imaging. Specifically, the calibration was performed as follows:

1. Single slice confocal scans were performed on free HL647 hydrazide in solution at 10 nM concentration increments, ranging from 0 nM to 60 nM.
2. A “volume element” was defined as a box of length 40 pixels, width 40 pixels, and height 1 pixel (or 1.6 µm by 1.6 µm by 1 µm).
3. The total fluorescence intensity of a volume element at each concentration was measured.
4. The average number of dye molecules present in a volume element at each concentration was calculated.
5. Total fluorescence intensity of a volume element versus the number of molecules present in a volume element was plotted for each concentration.
6. A line was fit to these data, and the slope represents the measured fluorescence intensity per molecule in the system (see Figure S5).

Calibration accuracy in determining oligomer size was confirmed by photobleach step counting, as discussed in Results.

### Single Molecule Confocal Laser Scanning Microscopy

Confocal laser scanning microscopy was performed on an Olympus FluoView 500 microscope mounted on an Olympus IX-71 frame. Images were acquired in line scan mode using an Olympus PlanAPO 60x, 1.42 NA oil immersion objective. The 488 nm line of an Argon laser was used to obtain FITC channel and DIC (Differential Interference Contrast) images, with illumination power of 140 µW, and a 633 nm HeNe red laser was used for HL647 excitation at a power of 28 µW. A BA660IF emission filter and Cy5 channel PMT settings of 850 volts, 6.0 gain, and 6% offset were used for all single molecule experiments. Scans were taken at medium speed (10.67 seconds per scan). Scans of a 43 µm by 43 µm field of view were taken at 1024 by 1024 pixel resolution, yielding an image plane pixel size of 42 nm by 42 nm. Axial resolution (full width at half-maximum in the z-direction) was set to 1 µm by adjusting the confocal aperture diameter to 590 µm. Imaging was performed at 37°C under 5% CO_2_ within 1 hour of exposure to Aβ40.

### Data Analysis in ImageJ

For live cell data, an outline representing the cell membrane was drawn around the inner edge of the cell's image in the DIC scan (see Figure S8). This outline was then overlaid on the fluorescence scan. All diffraction-limited fluorescence spots whose maxima fell on or within 1 µm outside of this line were designated as “cell-bound oligomers”. Irregularly shaped fluorescence spots, spots saturating the detector at four or more pixels, and spots associated with a clear non-membrane artifact (i.e., bigger than a resolution element) on the DIC image were rejected from the analysis. Remaining “cell-bound oligomers” were boxed with a 40 pixel by 40 pixel (approximately 1.6 µm by 1.6 µm) region of interest. The total photodetector output for such a square represents the intensity of a “volume element” containing the oligomer—a box 1.6 µm in length, 1.6 µm in width, and 1 µm in height with the oligomer at its center. The total fluorescence counts for this region of interest were recorded. An adjacent off-cell square of the same dimensions was designated as “local background”. The total counts for this box were then subtracted from the total counts present in the “oligomer” box to yield an integrated fluorescence intensity value for the oligomer (see Figure S5). Dividing this value by the intensity per dye molecule yields an oligomer size, in monomers, for each individual particle included in the analysis.

### DiO Labeling and Colocalization

For membrane colocalization studies on HL647cAβ40, cells were first incubated with or without 50 nM HL647cAβ40 in 1 mL media for 10 minutes. An aliquot of 6 µL of 1 mM Vybrant DiO cell-labeling solution (Invitrogen) was then added and cells were swirled to mix. Cells were incubated at 37°C prior to washing and imaging as described above. Colocalization analysis was performed in ImageJ using an open-source macro entitled Colocalization (written by Pierre Bourdoncle, available on the web at http://rsbweb.nih.gov/ij/plugins/). Pixels where the ratio of HL647 to DiO fluorescence intensity was greater than 40% were designated “colocalized” regions.

### Cell-bound Oligomer Quenching

For potassium iodide oligomer quenching, a 3 M solution of potassium iodide was prepared in HBSS and added onto cells to a final concentration of 300 mM. Cells were imaged before and after 2 minutes exposure to potassium iodide.

### Calcium Leakage Experiments

SH-SY5Y cells were loaded with fluorescent calcium indicator Fluo4-AM (Invitrogen) at room temperature for 15 minutes at a concentration of 1.7 µM. Cells were then incubated in HBSS for an additional 15 minutes prior to washing and loading into sampleholders. 50 µL of 1 µM unlabeled Aβ40 or HL647cAβ40 were added by pipette to 950 µL HBSS in the sampleholder, for a final concentration of 50 nM. To induce maximal calcium leakage, ionomycin in HBSS was added by pipette to a final concentration of 6 µM. Fluo4-AM ΔF for each frame *n* was calculated as:
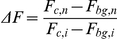



where *F_c,n_* represents cell body fluorescence in frame *n*, *F_bg,n_* represents fluorescence of an off-cell background region in frame *n*, *F_c,i_* represents initial cell body fluorescence, and *F_bg,i_* represents initial background fluorescence.

## Supporting Information

Figure S1 **Unlabeled Aβ40 and HL647Aβ40 cause dye to leak out of cell-derived blebs.** Blebs are prepared from rat basophilic leukemia (RBL) cells, as described in [49]. The blebs are first loaded with 5.7 µM calcein-AM. Blebs are then incubated on a kilned coverslip for 20 minutes, followed by gentle washing with buffer (10 mM HEPES, 150 mM NaCl, 2 mM CaCl_2_, pH 7.4). Imaging begins after another 10 minutes. Then, 45 minutes after the blebs are first imaged (green line), 200 nM HL647Aβ40 (red), 200 nM unlabeled Aβ40 (blue), or buffer (black) is washed over the coverslip. Average bleb fluorescence for each sample is plotted over time. Error bars represent the standard deviation of 3 to 6 single blebs for each sample.
(TIF)

Figure S2 **HPLC spectra for HL647cAβ40 and Aβ40.** The HPLC scan 215 nm absorbance spectrum is shown for 8 µM HL647cAβ40 (dark solid line, HL647AB40) and unlabeled Aβ40 (gray dashed line, UnlabAB40). Both peptides elute as a single peak with dimer-to-trimer molecular weight. An additional minor peak, visible in the void volume for the HL647cAβ0, may consist of peptide aggregates greater than 200 kDa; however, no fluorescent spots of the size and intensity expected for such large particles were observed in spin-coated samples examined by single molecule microscopy (see Figure S3). A limited number of large aggregates were detected in on-slide, in-solution samples, but these were a relatively small component (less than 10%) of the species observed.
(TIF)

Figure S3 **Confocal images of single HL647cAβ40 molecules.** The confocal image on the left depicts single molecules of dry HL647cAβ40. A droplet of 0.5 nM HL647Aβ40 in 10 mM sodium phosphate buffer, pH 7.4, was spin-coated onto a kilned glass slide. The confocal image on the right shows single molecules of HL647cAβ40 adhered to a glass slide after ten minutes' incubation of the slide with 50 nM peptide in media. Frames are 43 µm by 43 µm.
(TIF)

Figure S4 **Typical confocal mode photobleach traces for HL647cAβ40 diluted to 0.5 nM and spin-coated onto a kilned glass coverslip.** An example monomer trace is shown at top left; an example dimer is shown at top right. Example trajectories for two spots that did not photobleach in digital steps are shown at bottom left and right. Approximately 65% to 70% of particles bleached in clean, digital steps. Of these, 83% ± 3% bleached as monomers, and 15% ± 3% bleached as dimers. Of the particles that did not bleach in single steps, 5% ± 1% had intensities greater than was typical for observed monomers and dimers.
(TIF)

Figure S5 **Fluorescence intensity varies linearly with number of molecules in a volume element.** Total fluorescence intensity in a 40 by 40 by 1 pixel (1.6 µm by 1.6 µm by 1 µm) volume element versus expected number of HiLyte Fluor 647 hydrazide molecules present in that element. Intensity measurements were made at dye concentrations of 0 nM to 60 nM. Error bars represent the standard deviation for four different experiments. The slope of this line represents the fluorescence intensity of a single dye molecule.
(TIF)

Figure S6 **Example confocal image and TIRF photobleach trace for HL647cAβ40 trimers.** Example of a 40 by 40 pixel (1.6 µm by 1.6 µm) region of interest (ROI) in the confocal mode; this ROI contains a slide-bound HL647cAβ40 particle with the integrated intensity of a trimer (left). To the right is an example photobleach trace taken from a Total Internal Reflection Fluorescence (TIRF) film. Three discrete photobleach steps can easily be identified, marking the particle as a trimer.
(TIF)

Figure S7 **Potassium iodide quenches cytoplasmic fluorescence.** Confocal images of cells loaded with the cytoplasmic marker CellTracker™ Orange CMTMR (5-(and-6)-(((4-chloromethyl)benzoyl)amino)tetramethylrhodamine) (Invitrogen), before and after addition of potassium iodide to a final concentration of 300 mM (top row) or vehicle (Hanks' Balanced Salt Solution) (bottom row). The collisional quencher potassium iodide permeates the cell membrane quenches internal (cytoplasmic) fluorescence. Images are 43 µm by 43 µm.
(TIF)

Figure S8 **Particle identification for intensity-based oligomer size measurement.** Data analysis, from left to right: An outline was drawn onto the DIC image of each cell, just within the membrane. This outline was then pasted onto the corresponding fluorescence image. Edge-localized particles were identified as those spots whose maxima fell on or outside the outline and boxed with a 40 by 40 pixel region of interest. An adjacent off-cell 40 by 40 pixel region of interest was then identified as background.
(TIF)
