# Supplementary figures and images for: Direct Observation of Single Amyloid-β(1-40) Oligomers on Live Cells: Binding and Growth at Physiological Concentrations
Source: PLoS One. 2011 Aug 25;6(8):e23970. doi: 10.1371/journal.pone.0023970 (PMC3162019; doi:10.1371/journal.pone.0023970)

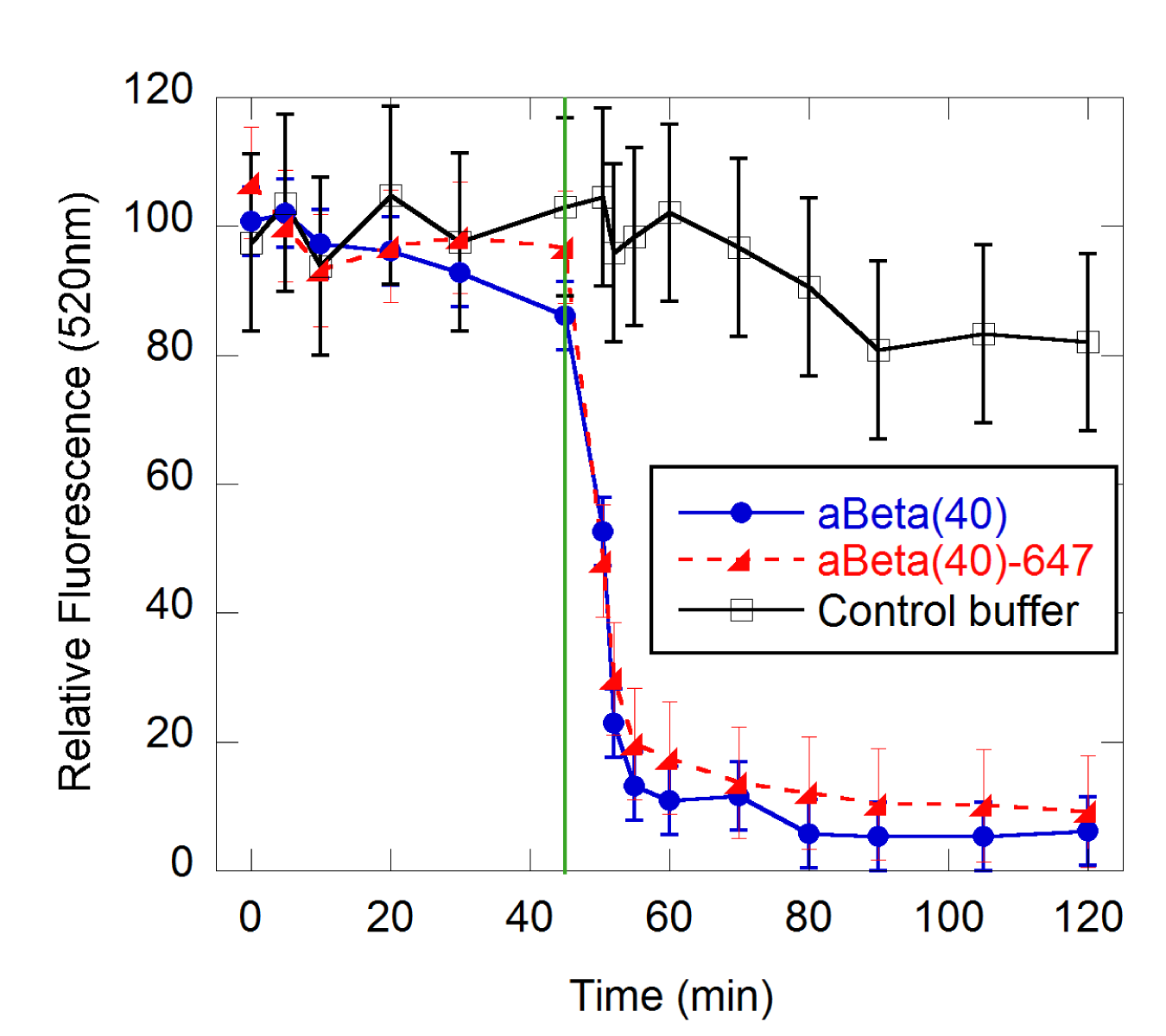

Supplement: Figure S1 — Unlabeled Aβ40 and HL647Aβ40 cause dye to leak out of cell-derived blebs. Blebs are prepared from rat basophilic leukemia (RBL) cells, as described in [49]. The blebs are first loaded with 5.7 µM calcein-AM. Blebs are then incubated on a kilned coverslip for 20 minutes, followed by gentle washing with buffer (10 mM HEPES, 150 mM NaCl, 2 mM CaCl2, pH 7.4). Imaging begins after another 10 minutes. Then, 45 minutes after the blebs are first imaged (green line), 200 nM HL647Aβ40 (red), 200 nM unlabeled Aβ40 (blue), or buffer (black) is washed over the coverslip. Average bleb fluorescence for each sample is plotted over time. Error bars represent the standard deviation of 3 to 6 single blebs for each sample. (TIF) [file pone.0023970.s001.tif]

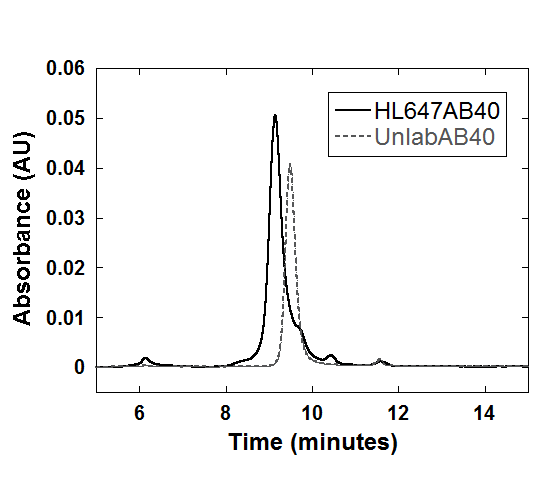

Supplement: Figure S2 — HPLC spectra for HL647cAβ40 and Aβ40. The HPLC scan 215 nm absorbance spectrum is shown for 8 µM HL647cAβ40 (dark solid line, HL647AB40) and unlabeled Aβ40 (gray dashed line, UnlabAB40). Both peptides elute as a single peak with dimer-to-trimer molecular weight. An additional minor peak, visible in the void volume for the HL647cAβ0, may consist of peptide aggregates greater than 200 kDa; however, no fluorescent spots of the size and intensity expected for such large particles were observed in spin-coated samples examined by single molecule microscopy (see Figure S3). A limited number of large aggregates were detected in on-slide, in-solution samples, but these were a relatively small component (less than 10%) of the species observed. (TIF) [file pone.0023970.s002.tif]

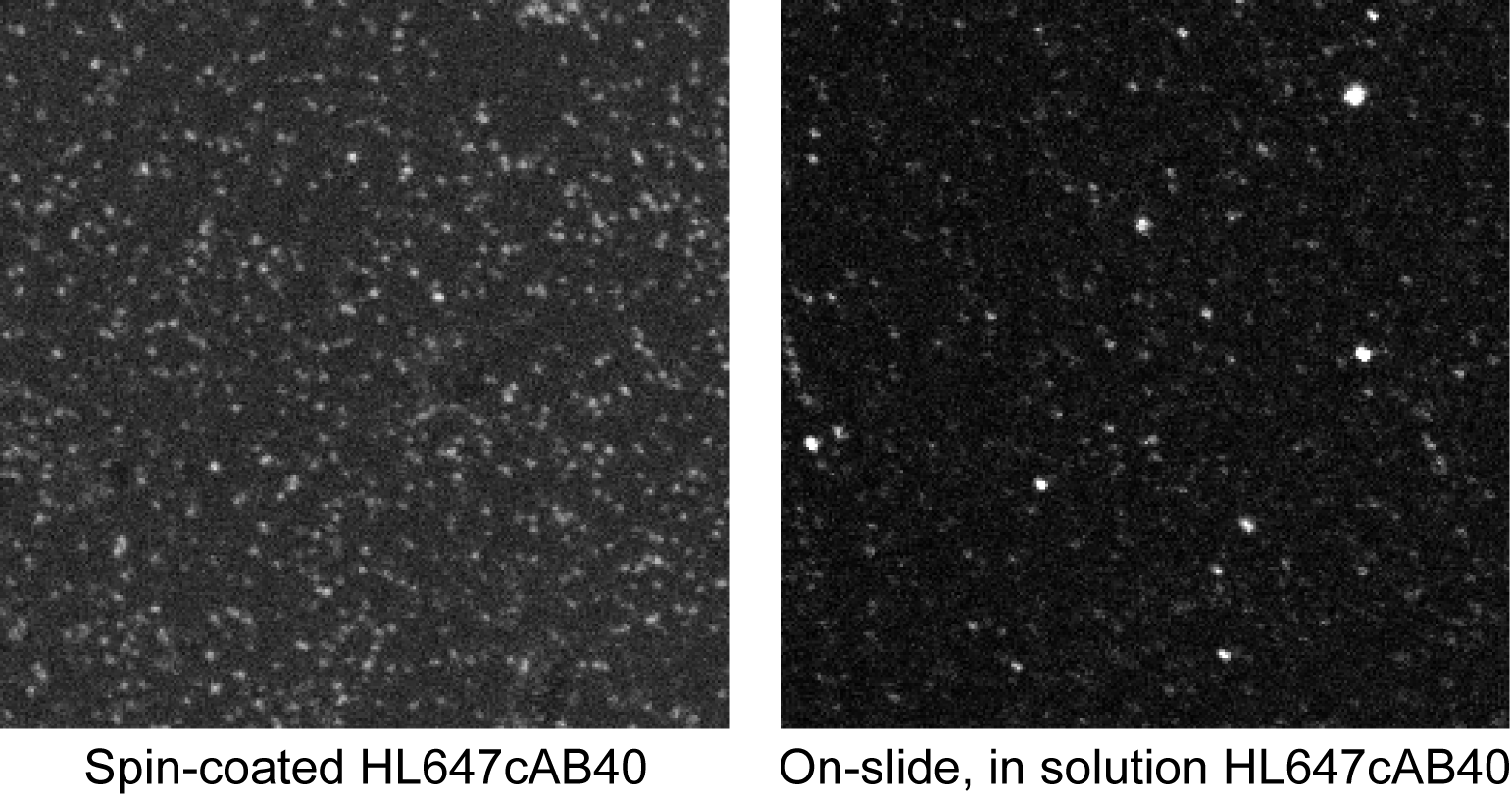

Supplement: Figure S3 — Confocal images of single HL647cAβ40 molecules. The confocal image on the left depicts single molecules of dry HL647cAβ40. A droplet of 0.5 nM HL647Aβ40 in 10 mM sodium phosphate buffer, pH 7.4, was spin-coated onto a kilned glass slide. The confocal image on the right shows single molecules of HL647cAβ40 adhered to a glass slide after ten minutes' incubation of the slide with 50 nM peptide in media. Frames are 43 µm by 43 µm. (TIF) [file pone.0023970.s003.tif]

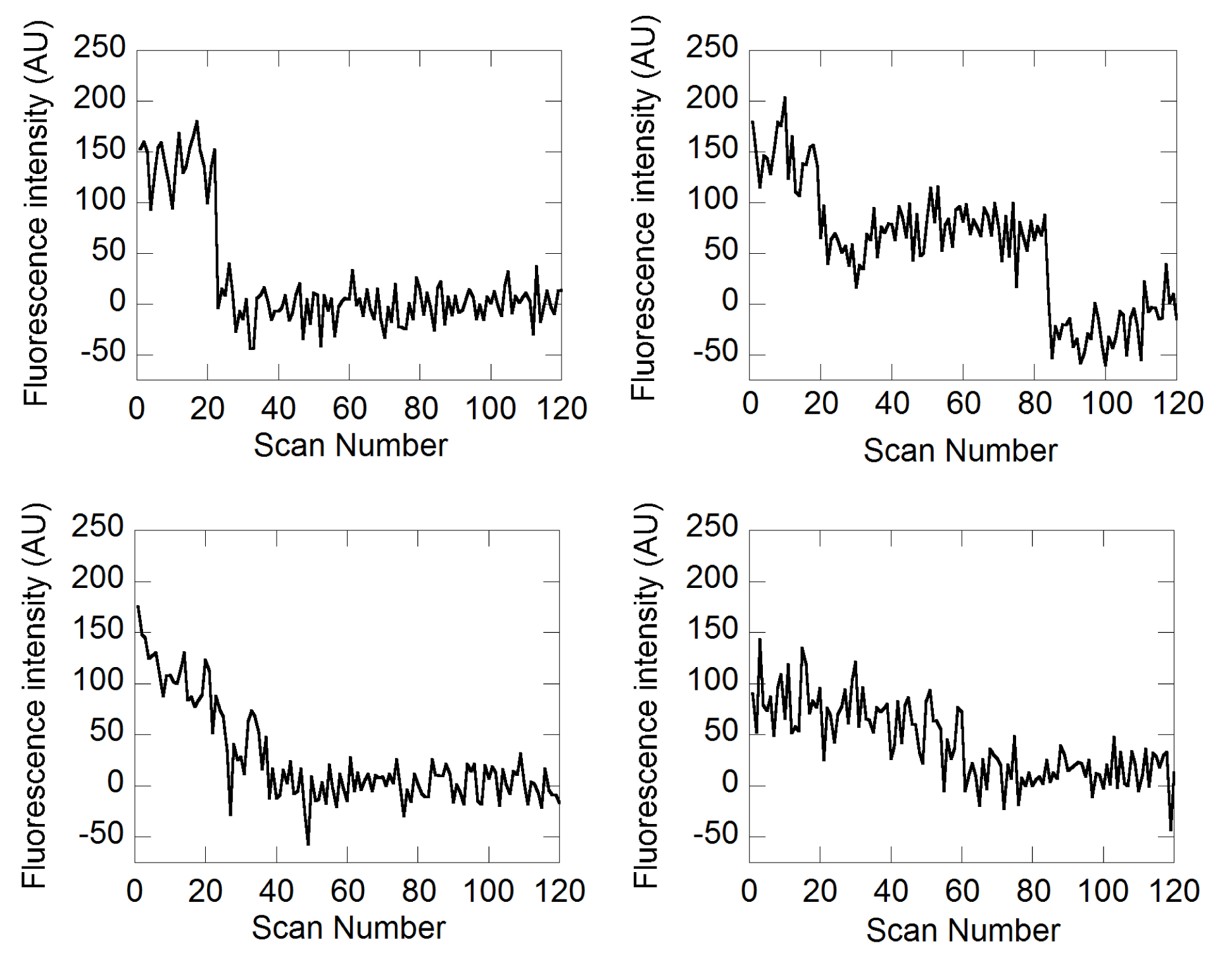

Supplement: Figure S4 — Typical confocal mode photobleach traces for HL647cAβ40 diluted to 0.5 nM and spin-coated onto a kilned glass coverslip. An example monomer trace is shown at top left; an example dimer is shown at top right. Example trajectories for two spots that did not photobleach in digital steps are shown at bottom left and right. Approximately 65% to 70% of particles bleached in clean, digital steps. Of these, 83% ± 3% bleached as monomers, and 15% ± 3% bleached as dimers. Of the particles that did not bleach in single steps, 5% ± 1% had intensities greater than was typical for observed monomers and dimers. (TIF) [file pone.0023970.s004.tif]

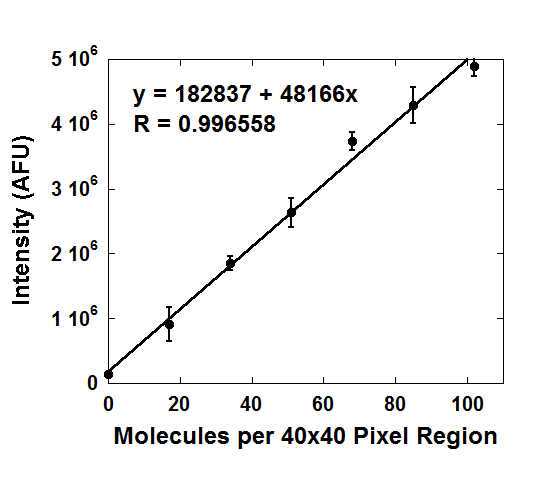

Supplement: Figure S5 — Fluorescence intensity varies linearly with number of molecules in a volume element. Total fluorescence intensity in a 40 by 40 by 1 pixel (1.6 µm by 1.6 µm by 1 µm) volume element versus expected number of HiLyte Fluor 647 hydrazide molecules present in that element. Intensity measurements were made at dye concentrations of 0 nM to 60 nM. Error bars represent the standard deviation for four different experiments. The slope of this line represents the fluorescence intensity of a single dye molecule. (TIF) [file pone.0023970.s005.tif]

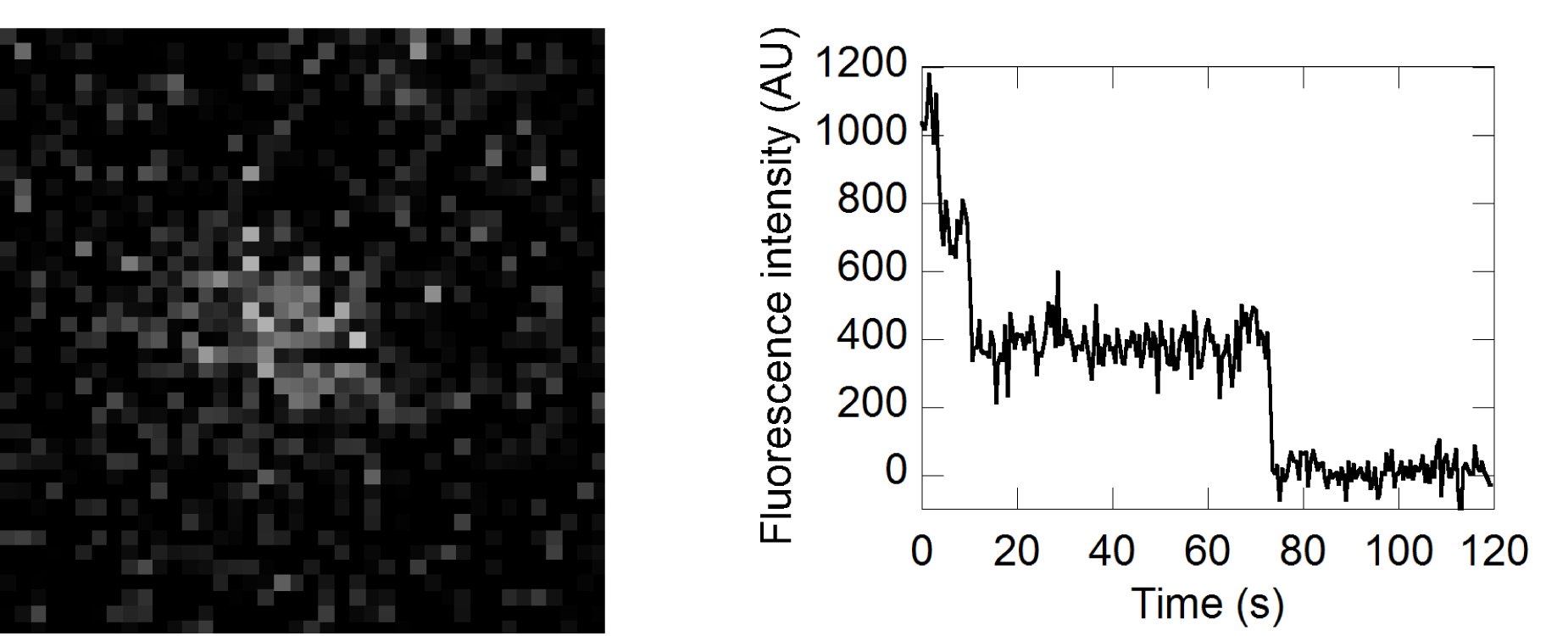

Supplement: Figure S6 — Example confocal image and TIRF photobleach trace for HL647cAβ40 trimers. Example of a 40 by 40 pixel (1.6 µm by 1.6 µm) region of interest (ROI) in the confocal mode; this ROI contains a slide-bound HL647cAβ40 particle with the integrated intensity of a trimer (left). To the right is an example photobleach trace taken from a Total Internal Reflection Fluorescence (TIRF) film. Three discrete photobleach steps can easily be identified, marking the particle as a trimer. (TIF) [file pone.0023970.s006.tif]

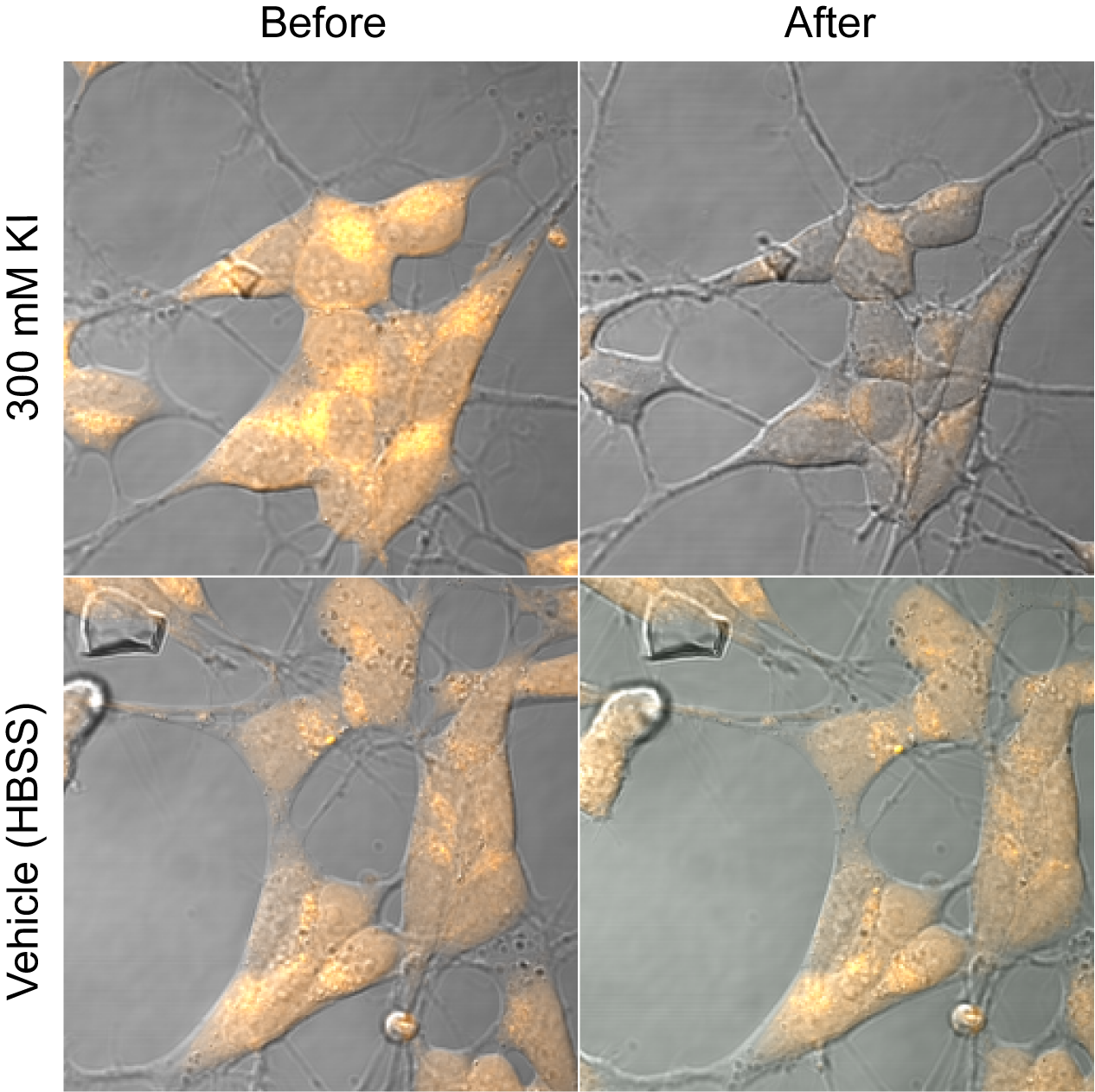

Supplement: Figure S7 — Potassium iodide quenches cytoplasmic fluorescence. Confocal images of cells loaded with the cytoplasmic marker CellTracker™ Orange CMTMR (5-(and-6)-(((4-chloromethyl)benzoyl)amino)tetramethylrhodamine) (Invitrogen), before and after addition of potassium iodide to a final concentration of 300 mM (top row) or vehicle (Hanks' Balanced Salt Solution) (bottom row). The collisional quencher potassium iodide permeates the cell membrane quenches internal (cytoplasmic) fluorescence. Images are 43 µm by 43 µm. (TIF) [file pone.0023970.s007.tif]

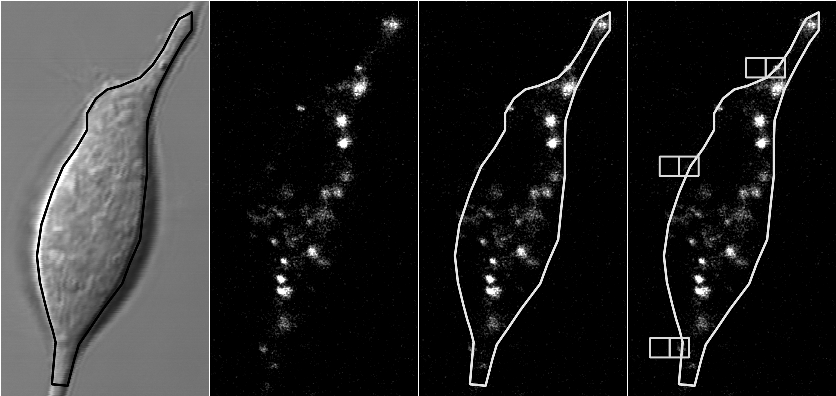

Supplement: Figure S8 — Particle identification for intensity-based oligomer size measurement. Data analysis, from left to right: An outline was drawn onto the DIC image of each cell, just within the membrane. This outline was then pasted onto the corresponding fluorescence image. Edge-localized particles were identified as those spots whose maxima fell on or outside the outline and boxed with a 40 by 40 pixel region of interest. An adjacent off-cell 40 by 40 pixel region of interest was then identified as background. (TIF) [file pone.0023970.s008.tif]
